# Additional Manufactured Interim Restorations: a Review on the Literature

**DOI:** 10.30476/DENTJODS.2021.91503.1589

**Published:** 2022-12

**Authors:** Marzieh Alikhasi, Zahra Jafarian

**Affiliations:** 1 Dental Research Center and Dept. of Prosthodontics and Implant, Dentistry Research Institute, School of Dentistry, Tehran University of Medical Sciences, Tehran, Iran; 2 Prosthodontist, Tehran, Iran

**Keywords:** Computer-aided design, Stereolithography, Digital technology, Prosthodontics

## Abstract

Interim restorations are essential in many clinical scenarios in which indirect restorations are administered. Additional manufacturing (AM) technology has recently been introduced and applied in different fields of dental practice. This study aimed to collect relevant information from published papers regarding different aspects of AM temporary restorations with a focus on the most relevant technical properties. An electronic search was performed on Medline/ PubMed/ Scopus databases up to April 2020 to find relevant, peer-reviewed articles about AM provisional restoration. Although promising results for AM temporary restorations were obtained, comprehensive application of this technology in making provisional restorations requires information to address the missing properties concerning the short time of application.

## Introduction

Interim restorations are inseparable parts of the dental procedure when indirect restorations are administered. These restorations help establish occlusal function and maintain esthetic requirements before final prosthesis insertion [ 1 - 2 ]. In dental implants, gradual loading of bone in the early time of maturation is accomplished by using interim implant-supported restoration [ 3 - 4 ]. If the shape, color, and contour of temporary restorations are satisfactory, they could be transferred to the dental technicians to copy these features to final restorations. Accordingly, the temporalization will assure the predictability of the final restoration contour [ 5 - 6 ].

The flexural strength, color stability, and hardness of the interim restoration must be considered especially when a long period of functioning is assumed, parafunctional habits are doubted, implant temporalization is planned, or long spam restorations are to be designed [ 7 - 9 ]. The interim restorations could be fabricated directly or indirectly [ 10 ]. Even though the indirect method requires extra clinical time and laboratory expense, it aids to have a more accurate restoration and lessens the risk of poor adaptation, existing excess monomer, and chemical and thermal harm to teeth and surrounding structures [ 11 - 13 ]. The conventional method in making indirect interim restorations using heat-cured or self-cured resin entails complicated laboratory processes [ 14 ]. 

Digital technology has an undeniable effect on prosthetic workflow and received popular significance in the dental industry [ 15 ]. CAD/CAM technology has recently been employed in the fabrication of indirect interim restoration with the major advantage of enhancing marginal and internal adaptation [ 5 , 16 ]. The digitally designed restorations could be incorporated into the subtractive process (milling) or additive manufacturing (AM) [ 9 , 17 ], which both could be done either in an office setting or dental laboratory [ 18 - 19 ]. The milling system which is the most common technique in fabricating indirect provisional restoration [ 20 ] was proven to have advanced mechanical properties in comparison to the direct intraoral technique [ 21 ]. As more standardized materials are used in milling technology than in conventional method, enhanced color stability, wear resistance, and marginal adaptation is observed in the milling technique [ 22 ]. Besides, the uniform structure of milled restoration, simple manufacturing procedure and a wide range of available materials and shades made milling the technique of choice in many clinical situations [ 23 ]. However, due to the subtractive nature of the milling process, limitation of bur size [ 24 ] and waste of extra material, which is claimed to be up to 90% [ 25 ], are reported. Moreover, an accurate micro-reproduction of the concave surface could not be achieved [ 26 ]. 

AM technology which is the most recent technological achievement in CAM procedure has made a tremendous effect on prosthetic dentistry due to being integrated with digital technology [ 20 ]. Using this method to produce prototype and cast pattern date back over 30 years [ 27 ]. Producing different prosthetic restoration [ 28 ], dental post and cores [ 29 ], dental models [ 30 ], patterns of casting restoration [ 20 , 28 , 31 ], surgical and radiographic guides [ 32 ], and occlusal devices [ 33 ] are among the AM application in contemporary dentistry. Seven different AM methods of stereolithography (SLA), material jetting (MJ), material extrusion or fusion deposition modeling (FDM), binder jetting, powder bed fusion, sheet lamination, and direct energy deposition have been ascertained by the International Society for Testing Materials (ASTM) [ 20 , 25 , 34 ]. Currently, SLA is the most used technique in all dentistry fields [ 9 , 34 - 36 ]. The user-friendly nature and the economical desktop size of equipment made the SLA technology wide established in the dental field [ 37 ]. Digital light projection (DLP) system possesses close similarity to SLA. The main difference is the source of light, which consist of small mirrors or arc lamp [ 38 ]. High resolution, low polymerization shrinkage, and smooth surface of printed objects are the reported characteristics of PolyJet technique for making interim restorations [ 39 ]. Temperature-controlled mask image projection based stereolithography (TCMIP-SL) and FDM technique are methods to make temporary restoration using high viscous composite resin [ 40 ]. 

AM is gaining enormous popularity over the milling method [ 35 ]. By using AM technology, large complex structures can be produced of different materials in a more economical way [ 41 ]. Forty percent reduction in waste material was reported by using AM instead of milling technique [ 25 ]. Moreover, depending on the size of printing objects and building platforms, multiple restorations could be fabricated simultaneously [ 9 , 20 ]. By reduction of the price and size of AM equipment and introduction of advancing methods which lead to utilizing this technology for a variety of dental material, AM technology has gained extended application in the dental field [ 42 ]. As there are a variety of methods for AM technology, there is strikingly lack of research to find the different parameters describing the characteristics of temporary restorations made by different AM systems [ 35 ]. This study aims to overview the articles assessing the different properties of AM provisional restorations and materials.

## Search Strategy

An electronic search in all languages was performed on MEDLINE/ PubMed/Scopus databases up to April 2020 to find relevant, peer-reviewed articles about AM provisional restoration and related issues such as strength, accuracy, wear performance, color, surface property, and design parameters. The abstracts of retrieved articles were assessed and the impertinent publications were discarded. An extra hand-search on the references of retrieved publications was conducted.

## Results

From the 55 identified articles, 31 articles were excluded, 5 case-reports, 14 in vitro studies evaluated the fabrication of AM single and multiunit copings, and 12 studies assessed different aspects of AM technology in other dental products. Twenty-four in vitro publications (twenty one in English and 3 in Korean languages) that evaluated the criteria of interim AM restorations and materials were chosen. As there were a variety of methods and study designs, no statistical analysis could be done. 

### Literature Review

The gathered information about AM temporary restorations are discussed in three main sections including restoration properties, fabrication parameters, and some other related issues. Accuracy, strength, wear behavior, surface roughness, and shade matching are discussed in restoration properties; build direction, layer thickness, support structure, cement thickness, and post-cure process are considered in fabrication parameters. The last part describes some other important issues related to AM interim restoration.

### Restoration properties

### Accuracy

Twelve publications evaluating the accuracy of AM interim restorations were found, seven on single-unit restorations, three on multi-unit restorations, one on implant-supported crowns, and one on material samples. The accuracy of interim restorations is mostly dependent on the method that was employed to fabricate them [ 43 - 44 ]. In the fabrication of interim restoration, a technology with the marginal and internal accuracy of at least 125 µm is mandated [ 9 , 45 ].

The discrepancy in AM technology could be the result of many complicated variants. Each printer has a resolution, reported by the manufacturer which could affect the final precision of the printed objects. It has been claimed that the printers have 25µm-29µm accuracy level [ 46 - 47 ]. The accuracy tolerance of 61 to 92µm between different AM technologies has been reported [ 48 ].

Moreover, the optical character of the photosensitive polymer could influence the process of polymerization and the accuracy. Due to the optical properties of each material, the light refraction leads to irradiation of the non-target area, which leads to increasing the size of a printed object in the most situations [ 49 ]. Other factor affecting the dimension of printed objects is designing steps and fabrication and the post-fabrication process [ 9 ]. 

In designing and printing stages, a set of factors can affect the accuracy including the software in use [ 50 ], build angle [ 35 , 51 ], printing layers number [ 52 ], number of printing objects [ 53 ], the thickness of layers, polymerization shrinkage [ 48 ], design of support structure [ 35 ], the property of light source [ 46 , 54 ], and post-printing process [ 34 ]. By determining the true dimensional change of each material and printer and the effect of build-direction and layer thickness on the accuracy, compensation on restoration design could be done to ovrcome the disparity [ 55 ].

In different AM systems, accuracy varies according to a variety of factors. In DLP system, lens quality, pixel size, platform resolution, depth of cure, and light intensity are among the influential variants [ 56 ]. Less deformation occurs during laser-assisted SLA, whereby this technology seems to fabricate more accurate restorations compared to DLP [ 51 ]. PolyJet technique had more accuracy in the proximal, marginal, and internal surface of interim crowns than the milled and molding methods. The high shrinkage of interim restoration made by the molding system caused the highest discrepancy compared to milling and AM methods. The mean marginal discrepancy of PolyJet and molding methods was reported 99 µm and 163 µm respectively. Meanwhile, PolyJet 3D-printing and self-cured temporary crowns showed significantly better occlusal adaptation compared to the milling group [ 44 ]. It can be rationalized by the inability of cutting bur to precisely mill the irregularity of occlusal surface [ 19 , 28 , 44 ].

Internal and marginal fitness of full-coverage single temporary restorations with different finish-line design fabricated using milling and 3D-printed SLA techniques were evaluated in an in vitro study [ 2 ]. Results of both fabrication methods were in the acceptable reported range. Knife-edge finish-line presented the least internal gap and rounded-shoulder-bevel design showed the least absolute marginal discrepancy [ 2 ]. Chamfer showed the largest absolute axial discrepancy in AM group. Since the knife-edge finish line is not an appropriate choice in many clinical scenarios, the rounded shoulder bevel finish line, due to possessing the least axial discrepancy and the lowest marginal gap (after knife-edge) was recommended by the authors [ 2 ]. The largest gap was found in the incisal area in both AM and milling techniques with the reported value of 169 µm and 209 µm, respectively which were about 1.5 and 1.8 times greater than the designed incisal cement gap [ 2 ].

A greater amount of material is needed for an implant-supported restoration compared to tooth-supported restorations due to more extensive tissue loss in implant restoration. Consequently, material shrinkage could cause higher discrepancy in implant interim restorations [ 4 , 6 ]; so could the multiunit restorations in which the mismatch between the printed restoration and CAD design seemed to be higher than in single crown [ 51 ]. The internal and marginal fit of implant-supported AM interim restorations showed to be better than conventional and milled techniques [ 4 ]. 

Contrary to the previous observation, Kang *et al.* [ 43 ] found better accuracy of milled PMMA restorations compared to SLA in both terms of precision and trueness. 

To justify this finding, variety of reasons based on the nature of AM technology could be stated. In Kang *et al.* [ 43 ] study milling process was done using a 5-axis machine compared to 3 axes stereolithography apparatus printer. Moreover, SLA technique comprises layer-by-layer deposition of photo-curable resin under UV light followed by polymerization stage, which may provoke inaccuracy mostly in mesiodistal direction [ 43 ]. Observing under the digital microscope revealed that the layered structure of printed restoration might result in less reproduction of designed surfaces in comparison to the milled group in both inner and outer areas. Another reason for the presence of inaccuracy in the outer surface of printed restoration was the trace of disconnected supporting elements from the outer surface [ 43 ]. In interim fabrication using a milling system, the same size of the designed restoration is milled in polymerized blocks, and no polymerization process is required. This minimizes the issue of material expansion or contraction during the milling process [ 57 ]. During the post-treatment process of AM restorations, deformation due to residual internal stress of the printed objects may occur. The last reason is light scattering in the manufacturing process. The output angle can also affect the precision of the prosthesis [ 58 ]. Sharp angles of a designed object lead to less accurate reproduction in PolyJet and fused deposition modeling techniques [ 59 ]. Based on the result of Lio *et al.* [ 60 ] study, sharp edges and areas with undercuts could be more accurately produced by milling system than by AM.

Another noteworthy aspect related to the accuracy of stereolithography technique is the laser light intensity, which is relied on penetrating the light beam through the AM material, the color and thickness of printed objects, and the refraction of light to the non-intended area. The darker material, the more intensity is required to polymerize a certain thickness. Grey, clear and white materials require the least intensity to be cured. Accordingly, the setting of the printing parameter should be done for each individual material [ 61 ]. In an in vitro study, different commercially available settings of printable material (white, black, grey, clear, tough, flex, and castable) were tested to find out the highest accuracy for further investigation [ 61 ]. The white resin had the least average percent error and was the most accurate testedmaterial [ 61 ] (Table 1).

**Table 1:** Demonstration of the results of available articles on accuracy of AM interim restorations

Accuracy	Restoration & Sample size	Groups	Measurement	Layer thickness	Printing orientation	Discrepancy	Method	Result
Dikova T. *et al.* [46] 2016	4-unit FPD* N#10	DLP**	Length, width, thickness	0.035 µm	NR***	35µm layer: smaller size (0.29%-1.1%).	Silicon replica	
				0.050 µm		50µm layer: larger size (1.51%-3.45%)		
Park JY *et al.* [4] 2016	Crown (implant)	DLP	Marginal fit	*NR	NR	Mean marginal discrepancy:	Silicon replica, Digital microscope	
	N#120	Milling				Conventional: 120.92 (±1.12)		
		Conventional				Milling: 58.02 (±19.75) mm		
						DLP: 56.85 (±22.24) mm		
						Overall discrepancy: DLP<Milling<Conventional		
Mai H.N. *et al.* [44] 2017	Crown N#36	PolyJetMilling Molding	Internal fit	5 µm	NR	Proximal contact discrepancy: Molding: 440(±351)µm, Milling: 271(±90)µm, PolyJet: 260 (±110)µm, Axial discrepancy:Molding: 87(±62)µm, Milling: 125(±30)µm, PolyJet: 139 (±23)µm	Image superimposition, Silicone-replica	PolyJet improved the fit of interim crowns in the proximal, marginal, and internal region
Lee W.S. et al [82] 2017	Crown N#30	Poly Jet SLA**** Milling	Internal and marginal fit	NR	NR	Mean discrepancy:PolyJet: 149.1µm, SLA: 91.1µm, Milling: 171.6µm	Silicone replica	AM techniques had better accuracy than milling
Tahayeri A. *et al.* [61] 2017	Bar	SLA	Length, width, thickness	100 µm	0, 15, 45, 90°		Digital caliper	The 90º orientation featured the best accuracy and minimize the required supporting structure
Alharbi N. *et al.* [2] 2018	Crown N#80	SLA Milling	Marginal fit Internal fit	0.05 mm	NR	Mean internal gap and marginal gap:SLA: 110 µm and 32 µm, Milling: 151 µm and 50 µm	Micro CT*****	SLA revealed lower marginal and internal discrepancy.
Kang S.Y. *et al.* [43] 2018	Crown N#22	SLA Milling	Trueness Precision	NR	180°	SLA Trueness:outer: 49.6±9. µm, inner: 22.5±5.1µm, Milling Turness:outer: 31.8±7.5µm, inner: 14.6±1.2µm, SLA Precision:outer 18.7±6.2µm, inner 26.9±8.5µm, Milling Precision:outer 25.4±3.1µm, inner 13.8±0.6µm	Best Fit	Accuracy of the milling system is superior compared to the AM technique
Dikova T.D. *et al.* [51] 2018	4-unit FPD N#14	SLA FDM****** DLP	Length, width, thickness	50 µm	0, 90°	FDM: smaller (0.10% -4.71%), SLA: bigger size of restoration (1.25% - 6.21%)	Caliper, Micrometer	SLA and DLP techniques are accurate in making temporary restoration.
Earar K. *et al.* [19] 2020	Crown N#20	Milling	DLP	Internal fit	20 µm	NR	Electronic digital caliper	No significant difference in trueness and precision were found
Choi J.W. [58] 2020	3-unit FPD N#30	Milling DLP SLA	Marginal fit Internal fit Precision	100 µm.	180º	Marginal and Internal Discrepancy, Milling: 48.1 and 101.8µm, DLP: 48.1 and 73.3µm, SLA: 37.6 and 69.9µm	Triple-scan	AM methods revealed better marginal and internal adaptation. Milling showed better precision

* FPD: Fixed partial denture, ** DLP: Digital light processing, *** NR: Not reported, **** SLA: Stereolithography, ***** CT: Computed tomography, ****** FDM: Fused deposition modeling

### Strength

Four publications were found which assessed the strength (flexural, compressive, and fracture strength) and hardness of AM provisional restoration materials [ 8 , 16 , 63 - 64 ]. Regardless of the aim of interim restoration administration, sufficient strength to endure occlusal force in a short or lengthy-time is required [ 6 , 19 ]. The occlusal load which seems to be exerted to dental restoration in the oral cavity is about 12 to 90N and the maximum load can reach to 909N [ 62 ]. Regarding the results of the studies on the strength of AM polymeric material, these restorations could not be applicable for a long-term period. However, their usage for interim restoration is justified [ 37 ]. Flexural strength of 50MPa has been recommended as a sufficient amount of strength for temporary restoration according to ADA-ANSI specification #27 [ 8 ]. 

Flexural strength of micro-hybrid composite SLA printed samples revealed to be lower than PMMA milled and heat-cured interim resin [ 8 ]. The layered structure of printed material predisposes these objects to crack propagation [ 35 ]. The fracture mostly occurred in the pontic area [ 63 ]. However, microhardness [ 8 ] and fracture strength [ 64 ] of SLA printed samples seems to be higher than milled and conventional groups. 

In a study in which the degree of conversion of printed resin samples was compared to self-cure temporary resin material, both groups were shown to be inhomogeneous in nature [ 61 ]. Printed samples were more polymerized in the area close to the printing platform [ 61 ] (Table 2).

**Table 2:** Demonstration of the results of available articles on strength of AM interim restorations

Strength	Restoration & Sample size	Groups	Measurement	Layer thickness	Printing orientation	Strength	Method
Digholkar S, *et al.* [8] 2015	Sample N#60	SLA* Milled Conventional	Flexural strength Micro-hardness	NR**	NR	Mean flexural strength:Printed: 79.54 MPa***, Milled: 104.20 MPa, Conventional: 95.58 MPa, Microhardness: Printed: 32.76 KHN****, Milled: 25.33 KHN, Conventional: 27.37 KHN	Universal testing machine, Microhardness tester
Alharbi N, *et al.* [16] 2016	Sample N#40	SLA	Compressive strength	0.05 mm	Vertical Horizontal	Compressive strength:Vertically printed: 297MPa, Horizontally printed: 257MPa	Universal testing machine
Park SM, *et al.* [63] 2017	3-unit FPD N#15	DLP*****	Flexural strength	NR	30°	Mean flexural strength:Methacrylate ester: 1119±305 N******, Bisphenol A: 619±150 N, Urethane acrylate: 413±65 N	Universal testing machine
Cho WT, *et al.* [64] 2019	Crown N#40	SLA DLP Milling Conventional	Fracture load Flexural strength	100 µm	0°	Mean flexural strength:SLA: 116.08±14.46 MPa, DLP: 146.37±7.25 MPa, Milling: 168.57±2.06 MPa, Conventional: 89.54±6.99Mpa, Mean fracture strength:SLA: 987.50±74.37 N, DLP: 1069.15±153.23 N, Milling: 748.49±135.61 N, Conventional: 678.48±152.16 N	Universal testing machine (fracture strength and 3-point bending test)

* SLA: Stereolithography, ** NR: Not reported, *** MPa: Megapascal, **** KHN: Knoop hardness number, ***** DLP: Digital light processing, ******: N: Newton

### Surface roughness

Two publications assessing the surface roughness of A-M provisional multi-unit restorations [ 51 , 55 ] and one publication on resin sample roughness [ 46 ] were found. Surface roughness seems to be affected by the type of material, technology to produce the restoration [ 51 ], layer thickness, and build angle [ 46 ]. Laser-assisted SLA leads to a rougher surface compared to the DLP [ 51 ]. 

FDM technology proved to have a less accurate and rougher surface than the SLA technology because of the nature of technique and properties of in used material [ 47 , 51 ]. The cubic samples fabricated by DLP stereolithography showed the lowest surface roughness with the object printed in the horizontal position (average value of Ra= 0.46-2.46µm), compared to the angled position (Ra= 1.74-2.77µm). The thicker layer and the less build inclination led to the rougher surface [ 46 ]. However, in another study assessing the surface roughness of 4-unit temporary restoration made by DLP technology, layer thickness seemed to be out of proportion to surface roughness [ 55 ]. The surface roughness of temporary restorations could be smoothed by mechanical finishing and polishing procedures [ 51 ] (Table 3).

**Table 3:** Demonstration of the results of available articles on surface roughness of AM interim restorations

Surface roughness	Restoration & Sample size	Groups	Measurement	Layer thickness	Printing orientation	Roughness value	Method
Dikova T, *et al.* [55] 2016	4-unit FPD*	DLP**	Surface roughness	35 µm	NR***	Average value of Ra****: 1.78 µm	Profile meter
	N#3			50 µm			Optical microscopy
Dikova T, *et al.* [46] 2016	Sample	DLP	Surface roughness	50 µm	0, 45°	Average value of Ra: Horizontal samples: 2.69µm	Profilometer
	N#20					Inclined samples: 1.8µm	
Dikova TD, *et al.* [51] 2018	4-unit FDP	DLP	Surface roughness	50 µm	0, 90°	Average value of Ra: FDM: could not be measured	Profile meter
	N#3	SLA*****				DLP: 2.40µm	Optical microscopy
		FDM******				SLA: 2.97µm	

* FPD: Fixed partial denture, ** DLP: : Digital light processing, *** NR: Not reported, ****: Ra: Roughness average, ***** SLA: Stereolithography, ****** FDM: Fused deposition modeling

### Wear behavior

Two publications assessing the wear resistance of AM interim materials were found [ 66 , 68 ]. Although provisional restorations are not expected to stand for a long time in the oral cavity, their resistance to abrasion in the time of their functional performance is of high importance. Wearing the material leads to an unstable occlusal relationship, shortage of chewing ability, roughness of the occluding surface, higher microbial absorption, and unsatisfactory esthetic outcome [ 8 , 65 ]. Acrylic DLP and SLA interim materials possessed sufficient but insignificantly less wear resistance than the milled group, but higher abrasion resistance than conventional PMMA was observed [ 66 ]. In SEM observation, instead of uniform wear facet in the milled group, vertical cracks were seen in AM samples. It could be the result of the layering structure of AM material that lessens the strength of restoration against abrasive wear [ 66 ]. Regardless of lower flexural strength, the greater microhardness of AM material comparing to the milled group could be an important reason for comparable wear behavior of printed material [ 8 ]. In both milled and AM restoration materials, wear abrasion was significantly less than conventional provisional material, which proved to have less mechanical strength due to the nonhomogeneous structure [ 67 ].

In AM material inter-layer cohesion seems to be stronger than intra-layer adhesion [ 68 ]. The character of the abrader could affect the pattern of wearing. Using a metal abrader caused the inter-layer bond to tear apart. However, the facet areas which were in contact with zirconia abrader were relatively smooth [ 68 ] (Table 4).

**Table 4:** Demonstration of the results of available articles on wear behavior of AM interim restorations

Wear behavior	Restoration & Sample size	Groups	Measurement	Layer thickness	Printing orientation	Volume loss	Method
Park JM, *et al.* [68] 2018	Sample	DLP*	Volume loss	100 µm	0°	Median range of volume loss against zirconia abrader:DLP: 1.11mm3, Milling: 1.20mm3, Conventional: 1.06mm3	Chewing simulator against zirconia and metal abrader
	N#60	Milling				Median range of volume loss against metal abrader: DLP: 1.22mm3, Milling: 1.11mm3, Conventional: 1.06mm3	
		Conventional					
Ahn JJ, *et al.* [66] 2019	Sample	DLP	Volume loss	100 µm	0°	Mean value of volume loss: DLP: 1.507±0.853 mm3	Dual-Axis Chewing Simulator, CAD software and SEM***
	N#20	SLA**				SLA: 3.178±0.791 mm3	
		Milling				Milling: 1.349±1.070 mm3	
		Conventional				Conventional: 8.242±2.625 mm3	

* DLP: Digital light processing, ** SLA: Stereolithography, *** SEM: Scanning electron microscope

### Shade matching

One study, which assessed the color dimension of AM interim material compared to autopolymerizing resin, was found [ 70 ]. Replicating the shade of existing dentition is paramount in any restorative procedure [ 69 ]. The shortage of available material and shade color are important limitations of the AM system [ 64 ].

A recent in vitro investigation [ 70 ] which assessed color dimension of AM interim restorative samples made by SLA, DLP, and PolyJet techniques compared to two groups of conventional composite and acrylic resin interim restorative material, found no accordance between tested groups and the conventional autopolymerizing materials in all 3 CIELab color dimensions. Based on the results of this study, while using AM material as an interim restoration, color matching varies significantly over conventional composite or acrylic material and for each system [ 70 ]. Contrary to other groups, the color dimension of PolyJet samples could not be measured using spectrophotometry. The possible explanation is that the shade of the specimen was not in the range of tooth shade [ 70 ]. Some of the post-curing methods were revealed to affect the shade of printed restoration [ 37 ]. To prevent the foreseen complications related to shade matching, custom shade guide fabrication should be done for each printable material. To gain further insight into the shade of AM materials, more studies have to be done [ 70 ]. 

### Fabrication parameters

### Build direction

Four publications [ 16 , 36 , 37 , 72 ] on the effect of build direction on mechanical properties of AM temporary restoration were found, two on single crowns [ 36 , 72 ], one on three-unit restorations [ 37 ], and one on interim material samples[ 16 ]. The build angle in each AM system should be individually determined in a way that self-supporting design with the minimum needed support structure, minimum fabrication time, least shrinkage between the layers [ 71 ] and high level of accuracy and structural properties would be resulted [ 72 ]. Moreover, the build angle is one of the factors affecting surface roughness [ 46 ]. As the printed materials are fabricated layer by layer, they seem to be anisotropic in nature [ 35 , 37 ]. Moreover, the X-Z axes in printing objects had different dimensional changes compared to the Y-axis. Accordingly, the build orientation has a definite effect on mechanical properties and dimensional changes of printed restorations [ 55 ]. The build angle determines the supporting area and the number of layers. So, it will clearly affect the time needed in the AM process and post finishing and polishing steps [ 35 , 52 ]. 

Many factors should be considered in determining build direction. It has been shown that vertically printed material in which the layers are perpendicular to occlusal load possess significantly higher compressive strength and lower probability of crack propagation compared to horizontally printed samples [ 35 ]. However, to achieve this layer arrangement, 0º build direction to the horizontal plane should be chosen in which the supporting elements will be located in the inner surface of the crown. This will end in inaccuracy in fitting surface [ 35 ]. In another study [ 37 ], distal and buccal positioning of AM material on printer platform revealed the highest strength in all tested FDP samples. However, since in distal alignment more layers of printed material should be used, a longer printing duration is expected [ 37 ]. Altogether, as the buccal direction of restoration on the print platform has less time-consuming process and supporting structures will not be located in the occlusal surface, this orientation is preferred over others and recommended by the authors [ 37 ]. By detecting the dimensional errors of each AM system, compensation in the designing process related to building direction could be implemented to decrease the amount of size discrepancy [ 51 ] (Table 5).

**Table 5:** Demonstration of the results of available articles on effect of build direction on physical properties of AM interim restorations

Effect of build direction	Restoration & Sample size	Groups	Measurement	Layer thickness	Printing orientation	Measured parameters	Method	Result
Alharbi N, *et al.* [36] 2016	Crown	SLA*	Dimensional accuracy (Root mean square estimate value)	50 µm	90, 120, 135, 150, 180, 210, 225, 240 and 270 °	Root mean square estimate value (thin support): 90°: 0.027mm, 120°: 0.029mm 135°: 0.032mm, 150°: 0.030mm 180°: 0.031mm, 210°: 0.035mm 225°: 0.042mm, 240°: 0.035mm 270°: 0.036mm	Digital subtraction method	120º with thin supportive structure had the highest accuracy and self-supporting structure
	N#18					Root mean square estimate value (thick support): 90°: 0.038mm, 120°: 0.031mm 135°: 0.038mm, 150°: 0.034mm 180°: 0.033mm, 210°: 0.040mm 225°: 0.033mm, 240°: 0.035mm 270°: 0.035mm		
Osman RB, *et al.* [72] 2017	Crown	DLP**	Dimensional accuracy (Root mean square estimate value)	50 µm	90, 120, 135, 150, 180, 210, 225, 240 and 270 °	Root mean square estimate value: 90°: 0.072mm, 120°: 0.056mm 135°: 0.049mm, 150°: 0.055mm 180°: 0.064mm, 210°: 0.049mm 225°: 0.050mm, 240°: 0.051mm 270°: 0.061mm	Digital subtraction technique and deviation pattern on color map	135° had the most self-support orientation, best accuracy and most favorable deviation pattern
	N#9							
Reymus M, *et al.* [37] 2019	3-unit FPD*****	DLP	Fracture strength	NR***	Occlusal		Universal testing machine	AM**** group was comparable to milled and conventional groups. The distal and buccal position had the highest fracture load.
	N#195	Milling			Buccal Distal			
		Conventional						

* SLA: Stereolithography, ** DLP: Digital light processing, *** NR: Not reported, **** AM: Additional manufactured, ***** FPD: Fixed partial denture

### Layer thickness

Two studies [ 46 , 55 ] that evaluated the effect of layer thickness on temporary AM restoration properties were found. The layer thickness which is defined as Z-axis in AM technology is chosen based on the type of object being printed and the accuracy needed in the reproduction of fine details [ 36 , 73 ]. Whenever accuracy is a paramount goal, the minimum layer thickness is the optimum choice. In a situation where time and cost are essential factors, the higher layer thickness is opted [ 74 ]. Other factors to determine layer thickness in SLA technique are optical property and depth of cure of the photosensitive resin and adequacy of radiation [ 39 ]. In another word, layer thickness together with the optical property of printed resin, determine the quality of polymerization [ 75 ]. Different layer thickness results in a different amount of size discrepancy. It can be rationalized by the polymerization that is taking place in the whole thickness of a layer [ 55 ]. The layer thickness recommended by manufacturers is ranged between 15 to 150µm with a surface roughness of 35 to 40 µm Ra [ 76 ]. The elastic modulus of printed material with a different layer thickness (25, 50 and 100µm) was the same. However, the peak stress and laser intensity were highest in 100 µm layer thickness. So, the parameter of thickness could be used to optimize the property of printable material [ 61 ].

### Supporting structure

One publication evaluating the effect of supporting configuration on the accuracy of single unit SLA interim restoration was found [ 16 ]. One of the limitations of the SLA system is mandatory to support structures that require more consumption of material and post-printing finishing [ 77 ]. A limited number of supports could lead to high distortion after the final polymerization and consequently lessen the accuracy of printed objects [ 55 ]. On the other side, more than needed supporting sets result in more polishing process, which reduce the surface accuracy [ 61 ]. The support elements are mainly designed semi automatically. Horizontal area and surface with less than 45º orientation to the long axis must be supported [ 36 ]. In the beginning, the software determines the position and number of the support structures in the particular build angle and afterward, the supports which are located in the inner and undesirable surface of the restoration are omitted manually [ 72 ].

### Cement thickness

No published data about programming the cement space in AM provisional restoration is available. However, in DLP 3D-printed resin coping, 85 µm cement gap was proved to have the highest reproducibility [ 78 ]. Programming cement space in the design step is crucial to minimize the marginal gap caused by axial discrepancy [ 44 , 79 ]. As the mid-axial gap in temporary resin SLA restoration was 41µm, which was lower than the programmed cement gap, higher cement space in the axial wall was recommended to lower marginal discrepancy [ 2 ]. 

### Post curing process

In one study, the effects of the different post-curing processes were assessed [ 37 ]. From a practical point of view, all printed products need some supporting areas which have to be removed and polished after the printing process [ 61 ]. The post-curing process for each specific material should be accomplished based on the manufacturer's recommendation [ 34 ]. To wash out the extra monomer from the surface of restorations, washing under running water, centrifugal force, or using 96% ethanol or isopropanol in the ultrasonically activated bath is recommended. These processes are usually done before the final polymerization [ 37 , 44 ]. The initial curing process for most available AM material using in dentistry field is the 385 nm ultraviolet radiation [ 37 , 39 ]. To complete the polymerization process, 30 minutes of polymerization in an ultraviolet unit has been recommended [ 35 , 58 ]. This post-curing process has a major effect on the properties of photosensitive resin material and enhances mechanical and biologic characters of AM restorations [ 37 , 39 ]. In an in vitro study that assessed the effect of the post-curing process on the fracture load of DLP printed 3-unit interim restorations, all specimen revealed higher levels of fracture strength after the post-cure procedure [ 37 ].

In the chair-side application of printed provisional restoration, the time required in the post-curing process (1-2 hours) should be in mind in a single appointment workflow [ 61 ]. Inserting the restorations on the cast when final polymerization is finished, can minimize the torsion occurred in this stage [ 55 ].

### Other tested parameters

The mechanical properties of a printed object can be improved by adding nanofillers to the printable material [ 80 ]. The material inuse for the fabrication of provisional restoration is resin-based, whereby they are susceptible to water absorption [ 37 ]. Hence, aging may significantly affect the strength of the printed resin material and inhibit long-term usage of them in the oral cavity [ 37 ]. The AM methyl methacrylate resin material is shown to be more prone to artificial aging than the PMMA milling and conventional material [ 37 ]. It has been shown that the flexural strength of direct temporary material was in the highest values between 7 and 28 days after water absorption [ 81 ]. In AM methyl methacrylate resin material, decreasing the fracture load was seen after aging [ 37 ]. The elastic modulus and the peak stress of printed resin samples seem to be comparable to conventional self-cure temporary resin material [ 61 ]. 

## Discussion

Incorporation of CAD/CAM technology in interim restoration fabrication, permit using standardized material instead of time-consuming chairside temporalization [ 5 , 8 ]. Easy transfer of data to a dental lab and straightforward laboratory process made milling technology the technique of choice in interim restoration. However, exhaustion of time and material is of major disadvantages of milling technology [ 82 ]. As the AM technology has offered different options in manufacturing the restorations, using this method in dentistry is increasing. Layer by layer production of material reduces material waste [ 43 ] and speed up the process of large and complicated object fabrication [ 25 ]. Producing the interim restoration with special axial and occlusal contour is a routine dependable application of AM in dentistry [ 72 ]. 

Currently, SLA due to the proven accuracy, the high-finished surface of the product, high-speed printing process, and detailed reproduction ability is the most used AM technique in all dentistry fields [ 20 , 34 - 35 ]. In the FDM process, a wide range of materials with different flowability can be utilized. However, the low resolution, limited surface quality, and time-consuming process made the FDM technique, not a routine candidate for provisional restoration [ 44 ]. The other AM method, which requires several hours to make a temporary restoration, is the ink-jet process. The efficacy of this technology in this field is doubted [ 83 ].

Methyl methacrylate and composite resins are two common materials used in interim restoration fabrication [ 84 ]. Polymer-based composite possesses sufficient strength and enough esthetic requirements to be used as a long-term temporary restoration [ 85 ]. However, the high viscosity of dental composite made it difficult to be used in AM technology [ 11 ]. The method that has been introduced to make temporary restoration using high viscous composite resin is TCMIP-SL, which claimed to produce great strength restorations with high accuracy and fast fabrication rate [ 40 ].

In assessing accuracy, precision seems to be better in milling system than AM technology [ 19 , 58 ]. In milling technology, the accuracy of the fabricating process depends on the motion scope of the milling device and the cutting instrument size. Moreover, the properties of milled material can affect the final accuracy [ 41 ]. 

There are some ambiguities in design parameters and their effects on the properties of temporary printed restoration. Moreover, no information regarding the minimum thickness of the temporary AM restoration, size of the connector, the maximum number of pontics, and the methods of repairing or relining them are available. Another difficulty in the interpretation of data acquired from the published articles was the variety of utilized technologies, materials, and software which made comparing the results of them not straightforward [ 9 ]. Because of these limitations and the absence of a randomized clinical trial, data extrapolation to clinical utilization should be done cautiously [ 86 ]. Other investigations on AM temporary material properties concerning the short time of application like tensile and shear strength, fatigue strength, biocompatibility, and color stability should be done.

We reviewed the available articles to weigh particularly related parameters in AM systems to fabricate the provisional restoration. In many aspects, AM has been comparable to milling and conventional methods. However, the enormous implication of AM technology has not necessarily downplayed the role of the milling and conventional systems in a dental procedure. Due to the dense structure and highly cross-linked nature of the tested milling group, the subtractive method presented the highest strength among the traditional and AM interim restorations [ 8 ].

## Conclusion

Using AM technology for fabrication of interim restoration has been expedited by extensive application of chair-side system, less expensive equipment and revealing the physical, biologic and esthetic benefits of new material compared to the traditional methods. However, further investigation to find the different properties of interim AM restorations in different AM systems are required.

## Conflict of Interest

The authors declare that they have no conflict of interest.
